# Predicting COVID-19 infection among older Syrian refugees in Lebanon: A multi-wave survey

**DOI:** 10.1371/journal.pone.0359272

**Published:** 2026-10-01

**Authors:** Berthe Abi Zeid, Tanya El Khoury, Sawsan Abdulrahim, Hala Ghattas, Stephen J. McCall

**Affiliations:** 1 Department of Health Promotion, Education, and Behavior, Arnold School of Public Health, University of South Carolina, Columbia, United States of America; 2 Center for Research on Population and Health, Faculty of Health Sciences, American University of Beirut, Beirut, Lebanon; 3 Department of Health Promotion and Community Health, Faculty of Health Sciences, American University of Beirut, Beirut, Lebanon; University of Diyala College of Medicine, IRAQ

## Abstract

**Objectives:**

Older refugees, exposed to a cluster of biological and social vulnerabilities, are more susceptible to infectious disease outbreaks and their complications. This study developed a predictive model estimating self-reported COVID-19 infection risk among older Syrian refugees in Lebanon using social determinants of health. Additionally, it described the barriers to diagnostic testing among those who reported having had COVID-19 infection at least once.

**Methods:**

This prediction modeling analysis uses data from a longitudinal study conducted between September 2020 and March 2022. Syrian refugees aged 50 years or older living in households that received assistance from a humanitarian organization were interviewed by phone. Self-reported occurrence at least one COVID-19 infection was the outcome of interest. The predictors were identified using adaptive lasso regression. The apparent model performance and discrimination were presented using the calibration slope and the Area Under the Curve (AUC).

**Results:**

Of 2,886 participants (median [IQR] age: 56 [52 –62]; 52.9% males), 283 individuals (9.8%) reported a COVID-19 infection at least once. Six predictors of at least one COVID-19 infection were identified: living outside informal tented settlements, having elementary and preparatory education or above, having chronic conditions, not receiving cash assistance, being water insecure and having unmet waste management needs. The apparent model had modest discrimination (AUC = 0.621 [95%CI: 0.587 to 0.655]) and good calibration (c-Slope = 1.004 [95%CI: 0.704 to 1.304]). Nearly half of the cases were diagnosed through testing. The main reasons for not testing were perception that the tests were unnecessary (n = 91 [63.6%]) or inability to afford them (n = 46 [32.2%]).

**Conclusions:**

The identified predictors provide insight into characteristics associated with higher predicted risk of COVID-19 infection among Syrian refugees. However, given the model’s modest predictive performance and reliance on lasso tuning, further validation and refinement in other populations and settings are needed before implementation. Awareness campaigns, screening measures, and cash assistance may be associated with reduced risk of infection in future pandemics.

## Introduction

The COVID-19 pandemic represented a major global health emergency, resulting in more than 779 million confirmed cases and 7 million deaths worldwide, and had lasting effects on health systems and economies globally [1]. The pandemic exacerbated preexisting health inequalities, further marginalizing vulnerable communities by restricting accessibility to essential services [2]. Displaced populations have increased exposure to infectious diseases and are disproportionally impacted by ‘shocks’ such as the COVID-19 pandemic [3]. For instance, refugees face various difficulties such as overcrowding, shortages of hygiene products and personal protective equipment, limited access to healthcare services, fear of deportation, and cultural and language barriers [4]. In addition, humanitarian organizations worldwide faced constraints in delivering assistance because of containment measures, nationwide lock-downs, and funding shortages, which limited their ability to support and protect vulnerable populations [5]. These disruptions may have exposed refugee populations disproportionally to infection risk through reduced ability to self-isolate due to the need to receive a livelihood [4].

Lebanon hosts the highest number of refugees per capita, including 1.5 million Syrian refugees, of whom 831,053 are officially registered with the UNHCR [6]. During the pandemic, Lebanon faced multiple economic, political and health crises in Lebanon; in addition, thousands of Syrians have been forced to return to Syria, which created an environment of fear among Syrians living in the country, regardless of their refugee status [7]. These combined challenges deteriorated the living conditions of Syrian refugees and were associated with loss of livelihoods, thereby placing them at an elevated risk of infection and its impacts [4]. Despite their high susceptibility to COVID-19 infection, testing in this population remained limited with a major barrier being lack of affordability [8].

COVID-19 provided an opportunity to inform preparedness and anticipatory action for mitigating future pandemic threats. Understanding the characteristics of individuals who were most likely to become infected is crucial for designing effective preparedness and anticipatory actions in humanitarian settings, particularly during the early stages of future pandemics, when information is limited and rapid decision-making is essential. Prediction models based on available social and demographic characteristics could help humanitarian and public health actors to efficiently identify individuals most at risk of infection, enabling them to provide essential assistance for prevention, while allocating limited resources more equitably.

Previous studies have identified age, gender, educational attainment, marital status, housing conditions, socioeconomic status, and presence of chronic illnesses as predictors of COVID-19, alongside a broader set of biological and clinical factors (e.g., biomarkers) [9–11]. However, evidence on the predictive value of readily available social determinants among refugee populations is limited. In addition, to date, predictive models to assess the risk of infection among refugees or older adults in the Middle East and North Africa (MENA) region are scarce. This represents an important evidence gap because models relying on routinely obtainable characteristics may be particularly useful when clinical and diagnostic information is limited or unavailable, as is often the case during the early stages of a pandemic and in resource-constrained humanitarian settings.

Therefore, this study aimed to develop a predictive model to estimate the risk of at least one COVID-19 infection among older Syrian refugees in Lebanon using social determinants of health. It also aimed to describe the barriers to diagnostic testing among those who had COVID-19 infection. These findings contribute to health emergency preparedness efforts by informing approaches that can be used to identify populations at risk in future pandemics and can be adapted to other settings or infectious diseases.

## Materials and methods

### Study design and setting

This was a prediction modeling analysis using longitudinal data from a five-wave study that monitored the vulnerabilities of older Syrian refugees residing in Lebanon to COVID-19 over time. The reporting of this study complied with the Transparent Reporting of a Multivariable Prediction Model for Individual Prognosis or Diagnosis (TRIPOD) reporting guideline and Strengthening the Reporting of Observational Studies in Epidemiology (STROBE) reporting guideline [12,13].

### Sampling and study population

This study targeted Syrian refugees aged 50 years or older who were identified from a complete roster of beneficiary households of a humanitarian Non-Governmental Organization (NGO) [Norwegian Refugee Council]. Within the beneficiary sample listing, all households in Lebanon that received assistance from the humanitarian organization between 2017 and 2020 and included an age-eligible member were contacted by phone (n = 17,384). Adults aged 50 years or older were invited to participate in the study. In households where multiple eligible adults were present, one individual was randomly selected. Verbal informed consent was taken from all participants. An evaluation of capacity to consent for participants aged 65 years or older was conducted using 5 modified items from the University of California, San Diego, Brief Assessment of Capacity [14]. Further details regarding participants’ capacity to provide informed consent are provided elsewhere [15,16]. The same participants were contacted over the course of five waves, spanning from September 22, 2020, to March 14, 2022. The study population for the current analysis, as depicted in Fig 1, consisted of participants who completed the first, second, and fifth waves. Participants reporting COVID-19 infection before enrolment in the study were excluded.

**Fig 1:**
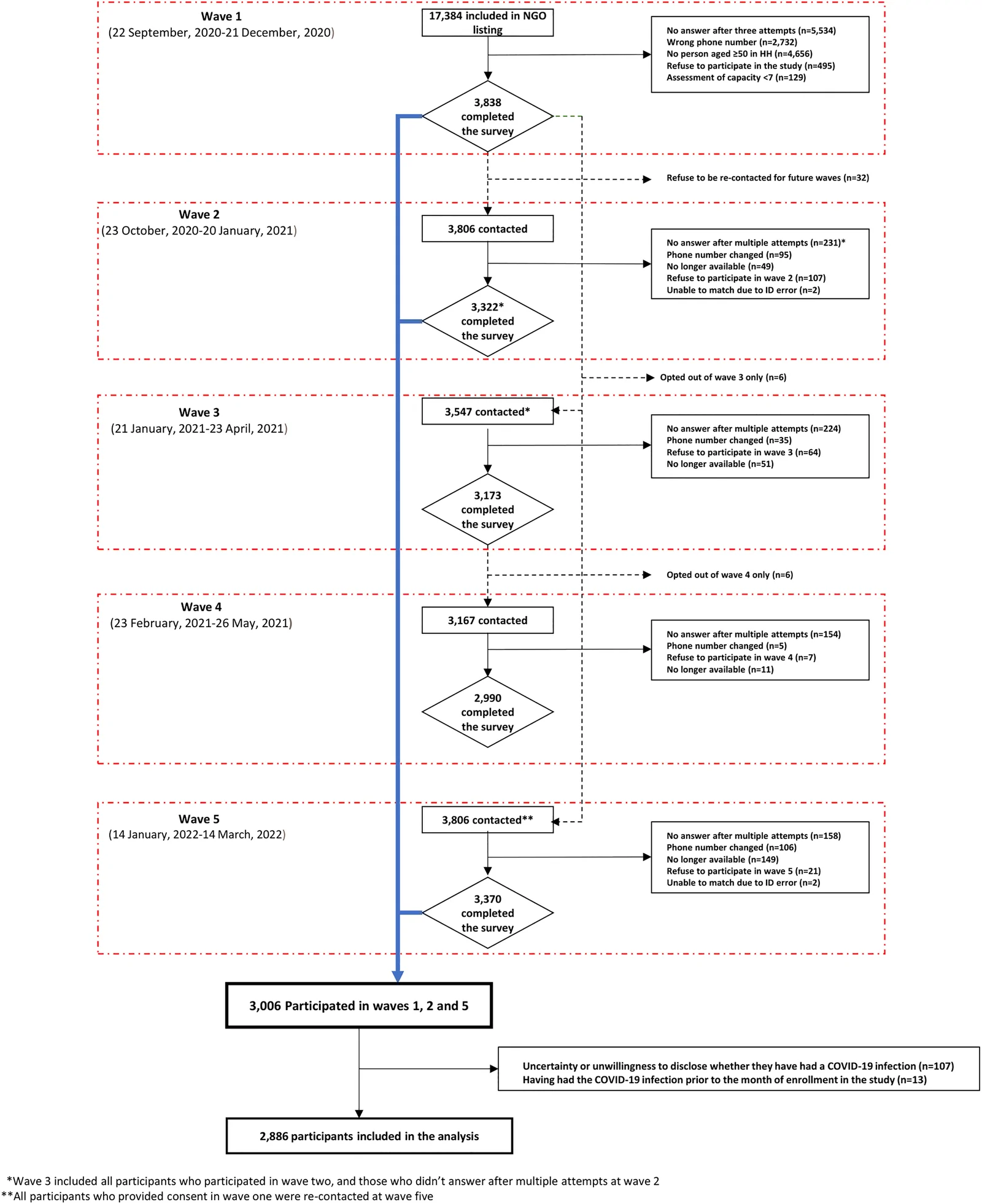
The flowchart of the study population.

### Data sources

The survey tool for each wave was created based on various sources, including established scales and context-specific questions, which resulted from a collaborative effort engaging academics, humanitarian workers, government representatives, and refugee community members. The questionnaire was drafted in English, then translated into Arabic. Before collecting data, the Arabic version was tested internally with data collectors and local community focal points to ensure comprehension. The survey was conducted in Arabic via computer-assisted telephone interview by trained data collectors, and responses were entered using Kobotoolbox. Daily monitoring was conducted simultaneously with data collection to ensure the quality of the data. Different sets of questions or modules were included at different waves. Additional information about the development of the survey tool can be found in previously published papers [16,17]. For the current analysis, demographic and health characteristics were extracted respectively from waves 1 (September 22-December 21, 2020) and 2 (October 23, 2020-January 20, 2021), whereas data on self-reported COVID-19 infection were obtained from wave 5 (January 14-March 14, 2022). None of the participants reported a COVID-19 infection between Waves 1 and 2; therefore, all predictors included in the prediction model were measured before the occurrence of the COVID-19 infection.

### Outcome measures

Self-reported occurence of at least one COVID-19 infection was the outcome of interest. In wave 5, participants were asked “Have you ever had COVID-19?”. Those who responded “Yes” were classified as having had COVID-19, irrespective of whether the infection had been confirmed by diagnostic testing. Participants who reported having had COVID-19 were subsequently asked whether their diagnosis was confirmed through a PCR or lateral flow test / rapid antigen, their reasons for not undergoing testing, date of their most recent infection and measures taken, which are detailed in the S1 Table. In a sensitivity analysis, we restricted the outcome to self-reported COVID-19 infections confirmed by a positive diagnostic test (PCR or lateral flow / rapid antigen test) to assess the robustness of the findings.

### Candidate predictors

The literature review guided the inclusion of the following candidate predictors for COVID-19 infection: age, sex, education, residence outside or inside informal tented settlements, marital status, number of self-reported physician diagnosed chronic conditions, household food insecurity (measured using the 8-item Food Insecurity Experience Scale (FIES); individuals are categorized as not food insecure if their score ranges between 0 and 3, and as food insecure if their score is 4 or higher) [18], household water insecurity (measured using the short-form Household Water Insecurity Scale) [19], having unmet waste management needs, receipt of cash assistance and having legal status documentation. The questions used to assess these predictors along with the corresponding survey wave from which they were obtained and their coding are provided in S2 Table.

### Missing data

The proportion of missing data was low for individual variables, not exceeding 3.5% for any variable. A total of 222 participants (7.7% of the total sample) had missing data for at least one variable included in the model. Given the low proportion of missing data and the observed missingness patterns, a complete-case analysis was considered appropriate [20].

### Statistical analysis

Descriptive analyses were conducted to summarize participants’ demographic and health characteristics, as well as COVID-19 infection characteristics, including the number of infections, confirmation through diagnostic testing (PCR or lateral flow / rapid antigen test), barriers to testing, and measures taken during the most recent infection (e.g., staying at home, mask use, use of traditional remedies, medication use, healthcare seeking, and pharmacy visits). Among participants who reported having had COVID-19, the timing of their most recent infection was summarized by calculating the percentage of participants reporting an infection in each month from November 2020 to March 2022. Frequencies and percentages were reported for categorical variables while median and interquartile range were reported for continuous variables. Unadjusted odds ratios (OR) and 95% confidence intervals (95% CI) for each potential predictor were assessed and reported by using unadjusted logistic regressions. A two-sided p-value below 0.05 was considered statistically significant.

All candidate predictors were categorical except age, which had a linear relationship with the outcome. Multicollinearity was assessed using variance inflation factors, all of which were below five, indicating no evidence of high collinearity among the candidate predictors. The available sample size was sufficient for model development based on the approach proposed by Riley et al. for multivariable prediction models with binary outcomes [21], as detailed in the S1 Methods.

All prespecified candidate predictors were entered simultaneously into an adaptive least absolute shrinkage and selection operator (Lasso) model, irrespective of their unadjusted p-values. The latter was implemented using the built-in Stata/SE 16 command “*lasso logit*” with the selection (adaptive) option. The adaptive lasso procedure first obtains preliminary coefficient estimates using a cross-validated lasso model, which are then used to construct predictor-specific penalty weights. Predictors with smaller initial coefficient estimates therefore receive greater penalization, allowing differential shrinkage across predictors. The penalty parameter (λ) was selected using 10-fold cross-validation [22], and the resulting penalized model was used for prediction. The penalized lasso coefficients were retained in the final prediction model. To obtain interpretable estimates of association, double-selection lasso logistic regression was performed using the “*dslogit*” command in Stata/SE 16 [23]. Double-selection lasso allows estimation of unpenalized coefficients for the variables of interest, which can be reported as ORs with robust 95% CIs. These ORs are interpreted as adjusted measures of association, conditional on the control variables selected through the double-selection procedure.

The discriminatory ability of the models was assessed using the Area Under the Curve (AUC), which ranges from 0.5 to 1.0. A value of 1.0 indicates a perfect ability to distinguish between those with and without the outcome, while a value of 0.5 suggests a discriminatory ability equivalent to chance. The calibration of models, which measures the agreement between observed outcomes and the model’s predictions, was evaluated through the C-Slope and calibration plot. The calibration plot categorizes individuals into 10 groups based on predictive probabilities and plots the mean predicted risk against the mean observed proportion of events. In a perfectly calibrated model, the plot would show a diagonal line with an intercept of 0 and a slope of 1. A C-Slope less than 1 indicates that the model has been over-fitted. Similarly, the quality of the prediction model is reflected by Brier score, ranging from 0 (indicating a perfect prediction accuracy) to 1 (indicating the worst prediction accuracy). Furthermore, calibration-in-the-large (CITL) was evaluated to determine the overall disparity between the observed frequency of events and the average predicted risk. The reported AUC, calibration slope, CITL, and Brier score were calculated on the same dataset used for model development and therefore represent the apparent model performance.

A sensitivity analysis was conducted by restricting participants to those with confirmed COVID-19 infection via diagnostic testing. All analyses were conducted using Stata/SE 16.

### Ethical approval

This study was approved by the American University of Beirut Social and Behavioral Sciences Institutional Review Board [Reference: SBS-2020-0329]. Verbal informed consent was obtained from all participants. Prior to participation, trained data collectors contacted potential participants by telephone, read the informed consent form aloud, answered any questions, and explicitly asked whether they agreed to participate. Participants were informed that participation was entirely voluntary, that they could withdraw at any time without any loss of benefits or services. The data collector documented the consent by signing the consent form, then sent to the participant via Whats App. Data collectors were trained and sensitized to the potential risks and vulnerabilities that refugee participants might encounter, including psychological distress. A referral list was compiled prior to data collection, and data collectors were trained on a clear referral protocol.

## Results

Out of the initial 17,384 households contacted during the first wave, 3,838 answered and were eligible, and a total of 3,322 Syrian refugees successfully completed both waves one and two. Among them, 3,006 participants also completed wave five. After excluding 120 participants who were uncertain or unwilling to disclose their history of COVID-19 infection or had experienced a COVID-19 infection prior to wave 1, the final sample for analysis comprised 2,886 participants (Fig 1). The median age of the participants was 56 (IQR = 52−62; range = 50−96); 1,527 (52.9%) were males and 1,761 (61.0%) lived outside tented settlements (Table 1). Among the 2,886 participants, 283 individuals (9.8% [95%CI: 8.7% to 10.9%]) reported experiencing at least one COVID-19 infection. The exact number of events across the categories of each candidate predictor is presented in Table 1.

**Table 1:** Characteristics of the study population and their association with the COVID-19 infection.

	Total (n = 2886)		At least one COVID-19 infection				Unadjusted Odds ratio (95%CI)
**No [n = 2603 (90.2%)]**		**Yes [n = 283(9.8%)]**	
**n**	**(%)**	**n**	**(%)**	**n**	**(%)**
**Age** median [Q1-Q3]	2886	56 [52–62]	2603	56 [52–62]	283	56 [53–62]	1.00 (0.98; 1.01)
**Sex**							
Male	1527	(52.9)	1377	(90.2)	150	(9.8)	*ref*
Female	1359	(47.1)	1226	(90.2)	133	(9.8)	0.99 (0.78; 1.27)
**Residence**							
Inside informal tented settlements	1125	(39.0)	1040	(92.4)	85	(7.6)	*ref*
Outside informal tented settlements	1761	(61.0)	1563	(88.8)	198	(11.2)	1.55 (1.19; 2.02)
**Marital status**							
Single, divorced or widowed	828	(28.7)	747	(90.2)	81	(9.8)	*ref*
Engaged or married	2058	(71.3)	1856	(90.2)	202	(9.8)	1.00 (0.76; 1.32)
**Education**							
Never attended school	1386	(48.2)	1276	(92.1)	110	(7.9)	*ref*
Elementary	751	(26.1)	669	(89.1)	82	(10.9)	1.42 (1.05; 1.92)
Preparatory and above	741	(25.7)	650	(87.7)	91	(12.3)	1.62 (1.21; 2.18)
*Missing*	*8*		*8*		*0*		
**Number of chronic conditions**							
0 chronic condition	875	(31.1)	812	(92.8)	63	(7.2)	*ref*
1 chronic condition	725	(25.8)	657	(90.6)	68	(9.4)	1.33 (0.93; 1.91)
2 or more	1212	(43.1)	1065	(87.9)	147	(12.1)	1.78 (1.31; 2.42)
*Missing*	*74*		*69*		*5*		
**Receiving cash assistance**							
Yes	2025	(70.3)	1840	(90.9)	185	(9.1)	*ref*
No	856	(29.7)	759	(88.7)	97	(11.3)	1.27 (0.98; 1.65)
*Missing*	*5*		*4*		*1*		
**FIES Food insecurity**							
Not food insecure	224	(8.1)	205	(91.5)	19	(8.5)	*ref*
Food insecure	2557	(91.9)	2306	(90.2)	251	(9.8)	1.17 (0.72; 1.91)
*Missing*	*105*		*92*		*13*		
**Household Water Insecurity Experiences Scale**							
No household water insecurity	1999	(69.5)	1826	(91.3)	173	(8.7)	*ref*
Household water insecurity	877	(30.5)	769	(87.7)	108	(12.3)	1.48 (1.15; 1.91)
*Missing*	*10*		*8*		*2*		
**Waste management needs status**							
Met	2369	(82.3)	2147	(90.6)	222	(9.4)	*ref*
Unmet	508	(17.7)	447	(88.0)	61	(12.0)	1.32 (0.98; 1.78)
*Missing*	*9*		*9*		*0*		
**Having legal status documentation**							
Not regularized	2172	(75.7)	1956	(90.1)	216	(9.9)	*ref*
Regularized	699	(24.3)	632	(90.4)	67	(9.6)	0.96 (0.72; 1.28)
*Missing*	*15*		*15*		*0*		

The bivariate analysis showed that living outside informal tented settlements (OR=1.55 [95%CI: 1.19 to 2.02]) compared to living inside, having elementary (OR=1.42 [95%CI: 1.05 to 1.92]) and preparatory education or above (OR=1.62 [95%CI: 1.21 to 2.18]) compared to never having attended school, having at least two chronic conditions (OR=1.78 [95%CI: 1.31 to 2.42]) compared to not having any, and experiencing household water insecurity (OR=1.48 [95%CI: 1.15 to 1.91]) compared to household water security were associated with increased odds of experiencing at least one COVID-19 infection. No statistically significant associations were found between the COVID-19 infection and age, sex, marital status, receiving cash assistance, household food insecurity, unmet waste management needs and legal status documentation (Table 1).

Six predictors of COVID-19 infection were identified including residence, education, number of chronic conditions, receiving cash assistance, household water insecurity and unmet waste management needs (Table 2). The apparent model had a C-Statistic of 0.621 (95%CI: 0.587 to 0.655), a calibration slope of 1.004 (95%CI:0.704 to 1.304), a brier score of 0.088 and CITL of 0.005 (95%CI: −0.121 to 0.131), which indicate modest discriminative ability and good calibration. Fig 2 shows the apparent calibration plot of the model and Table 2 presents the penalized coefficients, adjusted ORs, and their associated 95%CI.

**Table 2:** Predictive model of at least one COVID-19 infection.

Predictors (coding)	Coding	Penalized Coefficients*	aOR**	(95% CI)	P-value
**Residence**					
Inside informal tented settlements	0	0.37	*ref*		
Outside informal tented settlements	1		1.44	(1.08; 1.91)	0.012
**Education**					
Never attended school	0	0.23	*ref*		
Elementary	1		1.45	(1.02; 2.06)	0.036
Preparatory and above	2		1.64	(1.15; 2.35)	0.006
**Number of chronic conditions**					
0 chronic condition	0	0.27	*ref*		
1 chronic condition	1		1.34	(0.92; 1.95)	0.122
2 or more	2		1.74	(1.24; 2.44)	0.001
**Receiving cash assistance**					
Yes	0	0.17	*ref*		
No	1		1.21	(0.92; 1.59)	0.161
**Household Water Insecurity Experiences Scale**					
No household water insecurity	0	0.37	*ref*		
Household water insecurity	1		1.45	(1.11; 1.90)	0.006
**Waste management needs status**					
Met	0	0.23	*ref*		
Unmet	1		1.29	(0.94; 1.78)	0.109
**Constant**		−3.20			

*Lasso for prediction performed to report the penalized coefficients. As shown in the formula above, the predicted risk for an individual is obtained by multiplying each penalized coefficient by the corresponding variable coding for that individual and summing these values with the intercept.

**Lasso for inference was used to report the odds ratio and 95% confidence interval.

**Fig 2:**
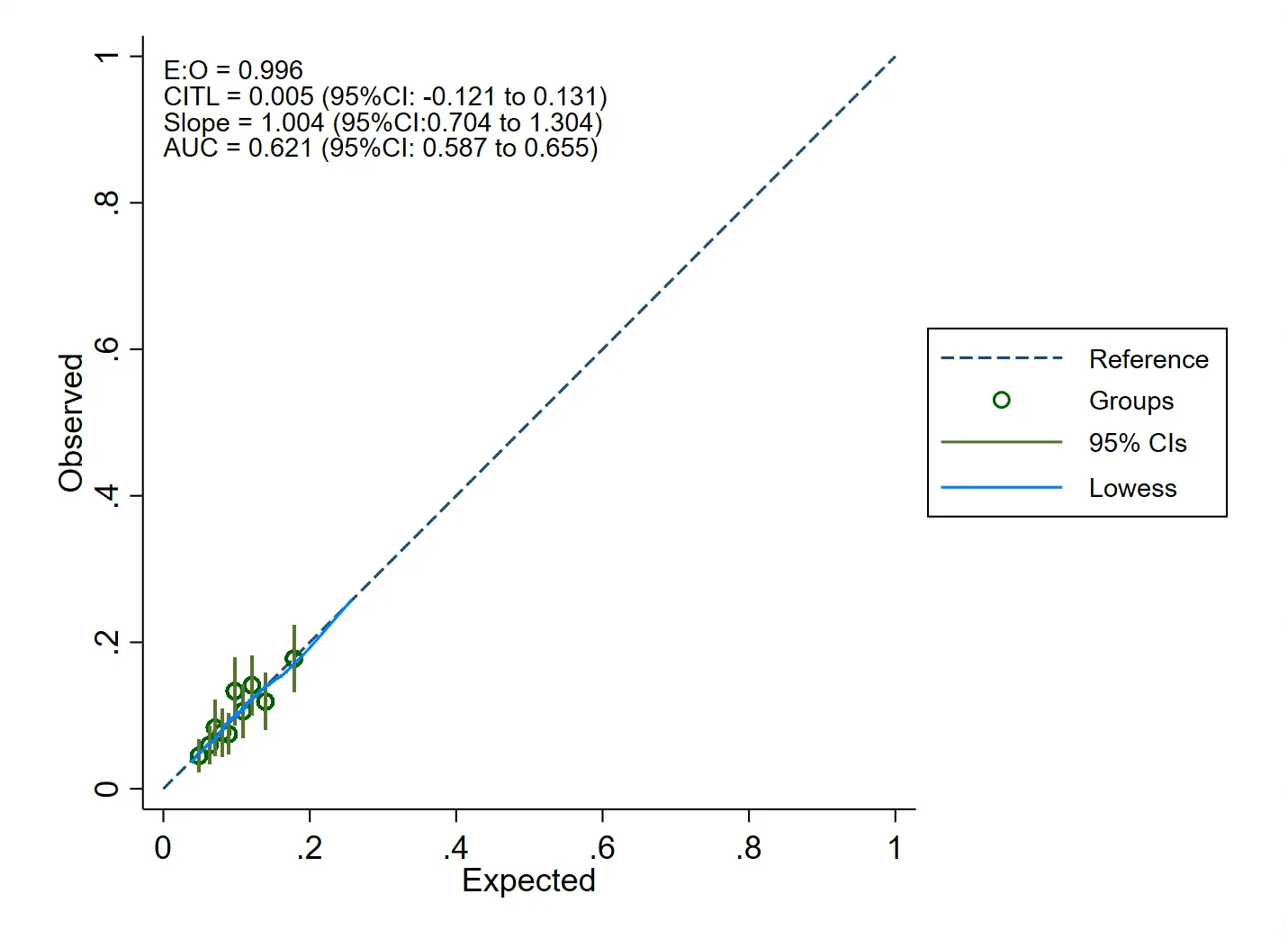
Calibration plot of the final model.

In a sensitivity analysis, the prediction model for confirmed COVID-19 confirmed based diagnostic testing included residence, education level, and the number of chronic conditions (S3 Table). In addition, its performance did not exceed that of the original model (C-Statistic: 0.628 [0.581 to 0.674] and C-Slope: 0.992 [0.587 to 1.397]) (S3 Table).

The predictors’ coefficients indicate that living outside informal tented settlements, having higher education, multi-morbidity, not receiving cash assistance, household water insecurity and having unmet waste management needs increased the risk of COVID-19 infection.

To illustrate, the predicted risk could be calculated using the formula below (Table 2):


$$\begin{array}{c} Predicted\;risk=\frac{e^{risk\;score}}{\text{1}+\;e^{risk\;score}}\;\times \text{1}\text{0}\text{0} \end{array}$$

Where,


$$\begin{array}{cc} isk\;score= & -\text{3}.\text{2}\text{0}+\text{0}.\text{3}\text{7}\;\times residency+\text{0}.\text{2}\text{3}\;\times education+\text{0}.\text{2}\text{7}\\ & \;\times Number\;of\;chronic\;conditions+\text{0}.\text{1}\text{7}\;\times receiving\;cash\;assistance\\ & +\text{0}.\text{3}\text{7}\;\times Household\;water\;insecurity\;scale+\text{0}.\text{2}\text{3}\;\times \;unmet\;waste\;management\;needs \end{array}$$

The predicted risk of having COVID-19 was 3.9% for a Syrian refugee who was living inside informal tented settlements, never attended school, did not have any chronic conditions, received cash assistance, was water secure and had waste management needs met. However, the predicted risk was 25.7% for an individual who was residing outside informal tented settlements, had preparatory education, had two chronic conditions, did not receive cash assistance, was water insecure and had unmet waste management needs.

Nearly half of participants who had at least one COVID-19 infection 138 (48.8%) reported being diagnosed through PCR or lateral flow / rapid antigen tests, 143 (50.5%) reported not being tested and 2 declined to answer. Among those who reported not being tested (n=143), the main reasons for not taking the tests were that they did not consider it necessary due to having COVID-19 symptoms (n = 91 [63.6%]), or they were unable to afford its cost (n = 46 [32.2%]) (Fig 3). No statistically significant associations were found between diagnostic testing and the participants’ characteristics (S4 Table).

In addition, among participants who reported at least one COVID-19 infection (n=283), 21 participants (7.4%) had the infection more than twice from the start of the pandemic until March 2022 (S5 Table). The most recent infection among participants occurred during the period of November 2020 to March 2022, with peaks during February 2021 (n = 30 [10.6%]), December 2021 (n = 32 [11.3%]), and January 2022 (n = 32 [11.3%]) (S1 Fig). During their last infection, 95.8% of participants stayed at home (n = 271; median: 3 weeks [IQR = 2; 4]), 90.8% wore a mask (n = 257), 79.2% used traditional remedies (garlic, ginger, anise tea) (n = 224), 73.1% took medication prescribed by the doctor (n = 207), 53.7% called (n = 152), 53.3% visited a doctor or healthcare center (n = 151) and 43.8% went to the pharmacy (n = 124) (S5 Table).

**Fig 3:**
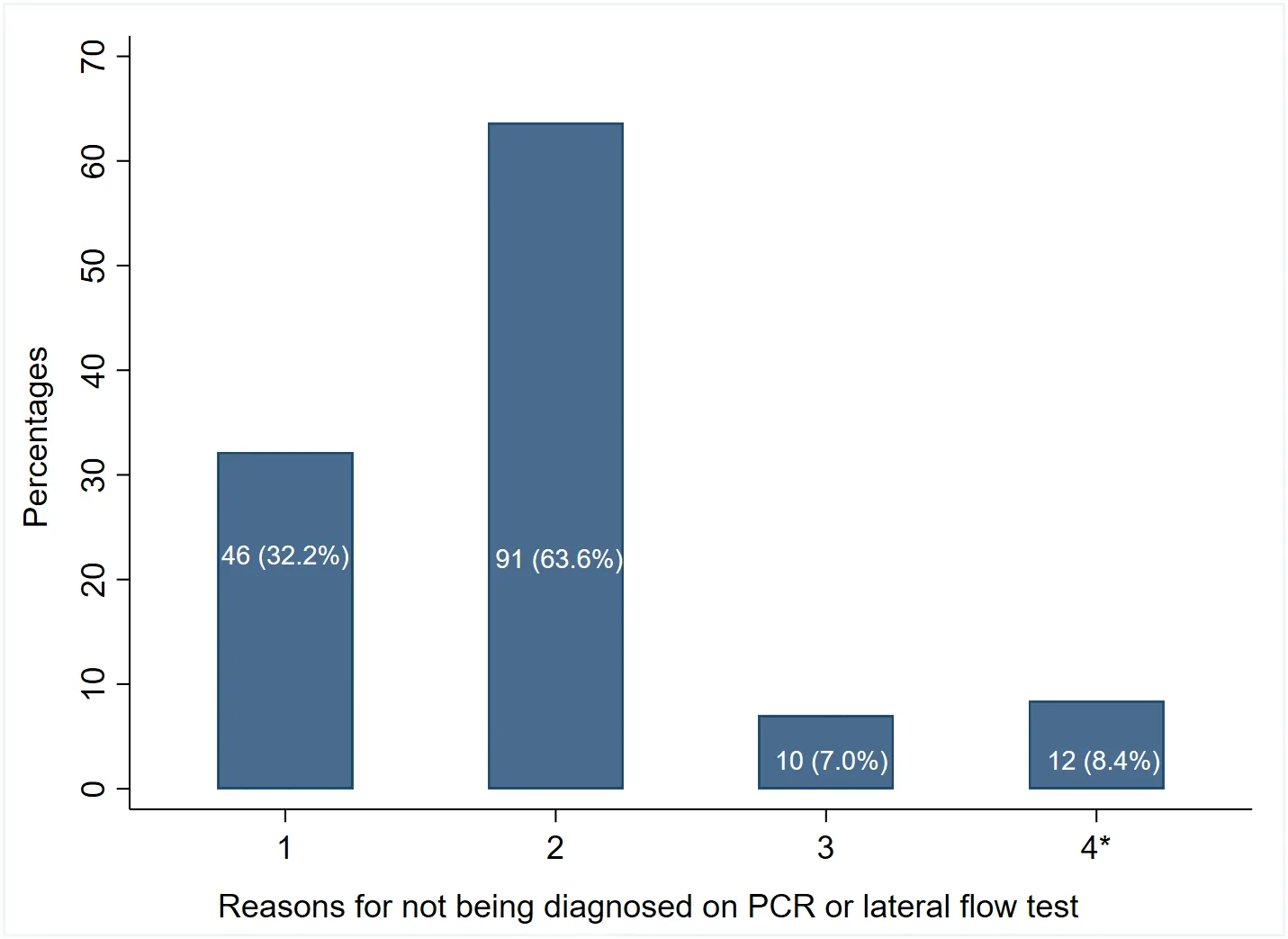
The reasons for not being diagnosed by PCR or lateral test / rapid antigen (n=143). Labels: (1) Unable to afford PCR test/lateral flow/ rapid antigen; (2) I didn’t think the test was necessary because I had COVID-19 symptoms; (3) I didn’t think the test was necessary because I had close contact with someone who had COVID-19; (4) Others; *Others include inability to access diagnostic testing center, fear of diagnosis and absence of recommendation from doctors or healthcare professionals.

## Discussion

From the start of the pandemic until March 2022, 9.8% of this population of older Syrian refugees (95% CI: 8.7% to 10.9%) had experienced at least one COVID-19 infection. The predictive model developed in this study identified six predictors of self-reported COVID-19 infection: residence, education, number of chronic conditions, receiving cash assistance, household water insecurity and unmet waste management needs. However, the model had modest performance. Nearly half of the cases were diagnosed through PCR or lateral flow / rapid antigen tests. The primary reasons for not taking the test were the perception that it was unnecessary due to having symptoms or the inability to afford it. Overall, there was high adherence to preventive measures during the period of infection, in addition to the consumption of prescribed medication or traditional remedies.

This study showed that the prevalence of COVID-19 infection among older Syrian refugees in Lebanon was 9.8%. A small-sample telephone survey similarly conducted in Lebanon by the Access Center for Human Rights, found that one-third of the 217 Syrian refugees contacted in April 2021 reported experiencing COVID-19 symptoms, most of whom were less than 44 years old [24]. In addition, two nationwide population-based cross-sectional studies reported the prevalence of COVID-19 infection in Lebanon [25,26]. The first study was based on a self-reported outcome and found a prevalence of 13.9% out of 1,119 participants, with a higher risk of infection among older adults [25]. The second study was based on lateral flow serological test and found a crude seroprevalence of 16.0%, with a slightly higher prevalence among those aged 60 years or older (18.5%). However, no statistically significant difference was found between the age groups [26]. The timeline of the most recent COVID-19 infection in this study was approximately aligned with the epidemic curve of confirmed cases [2]. These data showed that the prevalence of COVID-19 infection from our study among older Syrian refugees was lower than the national prevalence and may partly reflect an underestimation related to limited testing in this population or under-reporting due to fear of deportation or stigma as well as recall or social desirability bias. This pattern of under-representation of the true disease burden among displaced populations during pandemics was also observed in previous outbreaks beyond COVID-19 [27].

Globally, displaced populations frequently encounter individual, cultural, environmental, and financial barriers to access healthcare services [28,29], including testing for infectious diseases during pandemics [30,31]. Particularly, Syrian refugees in Lebanon faced barriers to diagnostic testing which were mainly related to individuals’ perceptions of lack of need or lack of affordability [9,25]. Although free testing services were available during the study period, complimented by a national effort to raise awareness [9], paid testing options also existed. Therefore, some participants may not have been aware of, or experienced difficulties accessing free testing services. Similarly, perceiving testing as unnecessary may have been related to mild or transient symptoms, perceptions of low personal risk, or limited awareness of the importance of confirming infection. In addition, perceived stigma, discrimination, fear of deportation, and limited accessibility to testing centers were reported as additional barriers to testing among Syrian refugees in Lebanon [9], and other displaced populations [30–34]. Despite these barriers, nearly half of the participants visited doctors, healthcare services or pharmacies after getting infected. Therefore, implementing COVID-19 screening in primary healthcare centers and pharmacies may be considered a potential strategy to improve case detection and control transmission among Syrian refugees. These settings are trusted sources and the front‐line of healthcare services. In addition, in future pandemics, given the use of non-prescribed medications and traditional remedies reported by older Syrian refugees, humanitarian organizations are encouraged to intensify their health promotion programs to increase awareness of testing availability and the importance of visiting a healthcare professional.

This study, focusing on older Syrian refugees in Lebanon, identified six predictors of at least one COVID-19 infection. These factors primarily pertain to socio-economic and health aspects, previously recognized in predictive models, such as education [10] and the number of chronic conditions [10,11]. Residence and receiving cash assistance emerged as unique factors within the refugee context. Notably, there is no prior research incorporating unmet waste management needs or water insecurity into prediction models, highlighting the added value of this study to the literature.

Contrary to previous findings, a higher level of education has been regarded as a protective factor against COVID-19 [35], while in this study, higher education was associated with increased odds of COVID-19 infection. During the pandemic, most Syrian refugees lost their jobs either permanently or temporarily, particularly those working in semi-skilled and unskilled labor such as agriculture and construction [36,37]. In contrast, individuals in more skilled occupations, which are typically associated with a higher education [36], were less likely to be laid-off. Consequently, one possible explanation for the observed association is that Syrian refugees with a higher education may have been more likely to have jobs requiring social interactions, potentially resulting in greater opportunities for exposure to COVID-19. Our exploratory analysis provides some support for this explanation. For instance, the proportion of participants who were working increased from 8.3% among those who had never attended school to 14.1% among those with elementary education and 14.8% among those with preparatory education or above (S6 Table). Furthermore, the interaction between education and work status was statistically significant, with participants with higher educational attainment who were working having higher odds of COVID-19 infection (result not presented). However, detailed information on the extent of interpersonal contact at work was not available to determine the attributable risk to occupations requiring greater social interaction.

Having chronic conditions was identified as another predictor of COVID-19 infection. A systematic review examining the risk factors of COVID-19 in migrant populations suggested the presence of comorbidities could be associated with a higher risk of infection [38]. One possible explanation for this association is that individuals with chronic conditions may visit healthcare centers and hospitals more frequently for disease management, therefore potentially increasing their risk of exposure to the virus [38], or make them more likely to be tested for and diagnosed with COVID-19.

Basic needs insecurities, such as water insecurity and unmet waste management needs, may contribute to the spread of infectious diseases including COVID-19 infection, through various pathways [10,39]. These include inadequate access to safe drinking water, drinking insufficient quantity of water, and limitations in practicing essential hygiene measures such as handwashing, bathing, and cleaning surfaces [40]. It is crucial to emphasize that the majority of Syrian refugees rely on humanitarian assistance to meet their essential needs, including water and sanitation [7]. Consequently, the absence of this assistance could worsen their living conditions and create opportunities for viral transmission within their households and communities.

Residence outside informal tented settlement was associated with higher odds of self-reported COVID-19 infection among Syrian refugees. This may partly be related to lower access to humanitarian assistance, as NGOs distributed hygiene kits, including masks and soap, specifically to refugees living in informal tented settlements, particularly during the early months of the pandemic [41–43]. Refugees living in host communities may therefore have had less access to such assistance [44], and reported lower receipt of cash and in-kind assistance (analysis not presented). Consequently, access to humanitarian programs including cash assistance, awareness campaigns, masks, hygiene kits and mobile clinics may reduce their risk of contracting the infection. These findings reinforce our previous recommendation, emphasizing the need for humanitarian organizations to redesign and expand their programs to ensure coverage for all refugees, including those living outside informal tented settlements, in order to address inequities and effectively control the pandemic [17].

Given the limited number and social aspect of determinants identified for predicting infection, the model may provide a simple approach for humanitarian organizations to identify refugees at a higher predicted risk of contracting the virus in future pandemics and outbreaks and therefore inform early interventions and allocation of resources. A major strength of this analysis is the temporal ordering of predictors and outcome, as predictors were measured in earlier survey waves, prior to the occurrence of COVID-19 infection. For instance, we intentionally used characteristics measured at baseline survey to evaluate whether information that could be easily collected early in an outbreak or obtained from routine assessments could predict COVID-19 infection.

The predictive performance of the model was limited. One possible explanation is the mis-classification of the outcome arising from reliance on self-reported COVID-19 infection. For instance, refugees might tend to under-report COVID-19 infection and overestimate adherence to preventative measures due to recall bias, social desirability bias or their fear of stigma and deportation. In addition, among participants who reported an infection, only 49% reported confirmation by PCR or lateral flow/rapid antigen testing, while the remaining infections were reported based on symptoms alone. Therefore, infections may have been missed due to asymptomatic or mild symptoms or may have been incorrectly attributed to COVID-19 because their symptoms overlap with those of other respiratory infections. Such outcome mis-classification would reduce the model’s ability to distinguish individuals who were truly infected from those who were not, as such, this may therefore contributed to the observed modest discrimination. Of note, the likelihood of recognizing and reporting an infection may have differed according to socioeconomic status, education, knowledge and attitudes related to COVID-19, access to healthcare and testing, and other individual or contextual factors. However, we were unable to assess the extent of such differential mis-classification, as infection and testing status were self-reported and data were collected remotely by telephone to comply with COVID-19 safety measures and minimize potential risks to participating refugees.

To explore the potential impact of outcome mis-classification, we conducted a sensitivity analysis restricting the outcome to test-confirmed infections. Only three predictors (residence, education, and chronic conditions) were retained, while performance remained modest. The reduction in the number of predictors suggests that predictor selection may be sensitive to how COVID-19 infection was defined and ascertained. However, the findings of the sensitivity analysis should themselves be interpreted cautiously. Restricting the outcome to test-confirmed infections substantially reduced the number of observed cases to 138, which may have limited statistical power. In addition, it could introduce selection bias if access to testing was non-random. Although no statistically significant differences in measured demographic and socioeconomic characteristics were observed between participants who reported infections with and without test confirmation, systematic differences in unmeasured factors affecting access to testing cannot be excluded. Future studies should therefore evaluate similar prediction models using confirmed outcomes, where feasible, ideally based on systematic diagnostic testing, to reduce outcome mis-classification and better assess the predictive value of the identified social determinants of health.

Furthermore, the study sample represents beneficiaries from a specific humanitarian organization and may not be representative of all Syrian refugees in Lebanon. Nevertheless, the study population shared several characteristics with Syrian refugee households included in the nationally representative 2021 Vulnerability Assessment of Syrian Refugees in Lebanon (VASyR), including a similar geographical distribution, with the largest proportions residing in the Bekaa and North regions, as well as comparable household debt, and receipt of eviction notices [45]. Further details on the characteristics and representativeness of the study population are provided elsewhere [16,46].

Considering these limitations, this model requires external validation before consideration for operational use in independent populations and diverse humanitarian settings. In addition, existing secondary data from previous major infectious disease outbreaks affecting displaced populations, such as cholera and measles could be used to explore the transferability of the modeling approach and assess whether similar social determinants have predictive value across different infectious diseases, epidemiological scenarios and contexts.

In conclusion, the identified predictors provide insight into characteristics predictive of higher risk of COVID-19 infection among Syrian refugees, particularly those who are required to work, seek healthcare or live outside tented settlements. The provision of humanitarian assistance may be associated with improved living conditions and reduced vulnerability to infectious diseases during pandemics. More broadly, prediction models that incorporate social determinants of health offer a rapid, low-cost, and practical approach for humanitarian and public health actors to identify at-risk individuals early and allocate resources more efficiently. The model developed in this study may provide a useful starting point for identifying socially vulnerable refugees at elevated risk during infectious disease emergencies, but external validation in other humanitarian settings and disease contexts is required. Following appropriate validation and refinement, integrating such models into emergency preparedness plans can strengthen anticipatory action and enable rapid targeting of vulnerable populations.

## Supporting information

S1 Table The survey questions on COVID-19 infection. (DOCX)

S2 Table The survey questions for the candidate predictors. (DOCX)

S1 Methods Sample size. (DOCX)

S3 Table Prediction model of COVID-19 infection, including participants diagnosed through diagnostic testing (n = 2741). (DOCX)

S4 Table Association between the characteristics of participants who had a COVID-19 infection and diagnostic testing. (DOCX)

S5 Table Description of participant behaviors following the COVID-19 infection. (DOCX)

S6 Table Educational level by work for pay during the last 7 days (wave 1). (DOCX)

S1 Fig Last occurrence of COVID-19 infection. (DOCX)
