# Supplementary material for: Predicting COVID-19 infection among older Syrian refugees in Lebanon: A multi-wave survey
Source: PLoS One. 2026 Oct 1;21(10):e0359272. doi: 10.1371/journal.pone.0359272 (PMC13630197; doi:10.1371/journal.pone.0359272)
Supplement: Supplementary file 1 [file pone.0359272.s001.docx]

# **S1 Table. The survey questions on COVID-19 infection.**

| **Have you ever had COVID-19?** | |
| --- | --- |
| - Yes - No - Don’t know - Refuse to answer | |
| **If yes, the following questions were asked** | |
| How many times have you had COVID-19? |  |
| When was the last time you have COVID-19? | Month / Year |
| Think of the last time you had COVID-19, when you found out, which of the measures did you take? *(please select all that apply)* ? | - Stay at home - Call doctor - Visit healthcare center or doctor - Go to the pharmacist - Wear a mask - Take medication prescribed by the doctor - Take medication on your own without prescription - Call UNHCR - Call MOPH hotline - Use traditional remedies (garlic, ginger, yensoon) - Other |
| - If other, specify | ……………………………. |
| - If stay at home – how long did you stay after you found out you had COVID-19? | Length of time:  Days…………….  Weeks………….. |
| If ever diagnosed with covid-19, was this diagnosed using either a PCR or lateral flow test? | - Yes - No - Don’t know / Refuse to answer |
| - If no, select the reason(s) why | - Unable to afford PCR test/lateral flow - Unable to access PCR testing center - I didn’t think the test was necessary because I had covid-19 symptoms - I didn’t think the test was necessary because I had close contact with someone who had COVID-19 - Fear of diagnosis - Other, Specify ………………………….. |
