# Supplementary material for: Predicting COVID-19 infection among older Syrian refugees in Lebanon: A multi-wave survey
Source: PLoS One. 2026 Oct 1;21(10):e0359272. doi: 10.1371/journal.pone.0359272 (PMC13630197; doi:10.1371/journal.pone.0359272)
Supplement: Supplementary file 2 [file pone.0359272.s002.docx]

**S2 Table. The survey questions for the candidate predictors.**

| **Possible predictors** | **Wave*** | **Questions** | **Variable coding** |
| --- | --- | --- | --- |
| Age | Wave 1 | Confirm participant’s year of birth | Continuous |
| Sex | Wave 1 | Confirm participant’s gender | 0: Male ; 1 : Female |
| Residency | Wave 1 | Confirms residence inside/outside ITS | 0: Inside ITS; 1 Outside ITS |
| Education | Wave 1 | Have you ever attended school?  If yes, what is your level of education? [elementary, preparatory, secondary, vocational, university and post graduate] | 0: Never attended school  1: Elementary school  2: Preparatory and above |
| Marital status | Wave 1 | What is your marital status? [single, engaged, married, separated/divorced, widowed] | 0: Single, divorced or widowed  1: Engaged or married |
| Number of chronic disease conditions | Wave 2 | Have you ever been told by a health care professional that you have any of the following chronic illnesses? (select all that apply): Hypertension, Diabetes, Cardiovascular Diseases, Chronic Respiratory Diseases, Rheumatoid Arthritis, Chronic kidney diseases and Cancer | 0: 0 chronic condition  1: 1 chronic condition  2: 2 or more |
| Food insecurity | Wave 1 | Food Insecurity Experience Scale^1^ | 0: Not food insecure  1: Food insecure |
| Water insecurity | Wave 1 | Household Water Insecurity Experiences Scale^2^ | 0 : No household water insecurity  1: Household water insecurity |
| Unmet waste need | Wave 2 | Do you have an unmet waste need? | 0: Met  1: Unmet |
| Receipt of cash assistance | Wave 1 | Have you received any cash assistance in the last 6 months? | 0: Yes  1: No |
| Regularization | Wave 2 | Do you have regularized residency in Lebanon? | 0: No  1: Yes |

* Wave 1: September-December 2020; Wave 2: October 2020 – January 2021

1 Cafiero C, Viviani S, Nord M. Food security measurement in a global context: The food insecurity experience scale. *Measurement.* 2018;116:146-152.

2 Young SL, Miller JD, Frongillo EA, Boateng GO, Jamaluddine Z, Neilands TB. Validity of a four-item household water insecurity experiences scale for assessing water issues related to health and well-being. *The American journal of tropical medicine and hygiene.* 2021;104(1):391.
