# Supplementary material for: Predicting COVID-19 infection among older Syrian refugees in Lebanon: A multi-wave survey
Source: PLoS One. 2026 Oct 1;21(10):e0359272. doi: 10.1371/journal.pone.0359272 (PMC13630197; doi:10.1371/journal.pone.0359272)
Supplement: Supplementary file 3 [file pone.0359272.s003.docx]

# **S1 Methods: Sample Size**

We specified an outcome prevalence of 9.8%, 13 candidate predictor parameters derived from 11 candidate predictors, and an anticipated C-statistic of 0.70, representing a prespecified minimum acceptable level of model discrimination^[1]^. The required sample size was calculated using the “*pmsampsize*” command in Stata/SE 16, which converted the specified C-statistic and outcome prevalence into an anticipated Cox–Snell R² of 0.0466 - reflecting the expected overall predictive strength of the model - and targeted a shrinkage factor of 0.90 - allowing no more than 10% shrinkage of the predictor effects to minimize overfitting. The required sample size required was 2,445 participants, corresponding to approximately 240 outcome events and 18.43 events per predictor parameter (EPP). The available complete-case sample exceeded the estimated minimum sample size required for prediction model development.

1. De Hond, A. A. H., Steyerberg, E., & Van Calster, B. (2022). Interpreting area under the receiver operating characteristic curve.. *The Lancet. Digital health*. https://doi.org/10.1016/s2589-7500(22)00188-1
