# Supplementary material for: Predicting COVID-19 infection among older Syrian refugees in Lebanon: A multi-wave survey
Source: PLoS One. 2026 Oct 1;21(10):e0359272. doi: 10.1371/journal.pone.0359272 (PMC13630197; doi:10.1371/journal.pone.0359272)
Supplement: Supplementary file 4 [file pone.0359272.s004.docx]

# **S3 Table. Prediction model of COVID-19 infection, including participants diagnosed through diagnostic testing (n=2741)**

| **Predictors** | **Lasso for prediction*** | | **Lasso for inference**** | | |
| --- | --- | --- | --- | --- | --- |
|  | **Codes** | **Coefficients** | **OR** | **(95% CI)** | **P-value** |
| **Residency** |  |  |  |  |  |
| Inside informal tented settlements | 0 | 0.46 | *ref* | | 0.035 |
| Outside informal tented settlements | 1 |  | 1.53 | (1.03 ; 2.28) |  |
| **Education** |  |  |  |  |  |
| Never attended school | 0 | 0.15 | *ref* | |  |
| Elementary | 1 |  | 1.67 | (1.04 ; 2.68) | 0.034 |
| Preparatory and above | 2 |  | 1.62 | (0.97 ; 2.69) | 0.064 |
| **Number of chronic conditions** |  |  |  |  |  |
| 0 chronic condition | 0 | 0.43 | *ref* | |  |
| 1 chronic condition | 1 |  | 0.99 | (0.55 ; 1.77) | 0.980 |
| 2 or more | 2 |  | 2.01 | (1.24 ; 3.25) | 0.004 |
| **Constant** |  | -3.89 |  |  |  |
| **Model performance** | **Estimates** | **[95% CI]** |  |  |  |
| C-statistic | 0.628  (0.581; 0.674) | |  |  |  |
| C-slope | 0.992  (0.587; 1.397) | |  |  |  |
| CITL | 0.017 (-0.155; 0.190) | |  |  |  |

*Lasso for prediction performed to report the coefficients used to calculate the predicted risk

**Lasso for inference was used to report the odds ratio and 95% confidence interval
