# Supplementary material for: Predicting COVID-19 infection among older Syrian refugees in Lebanon: A multi-wave survey
Source: PLoS One. 2026 Oct 1;21(10):e0359272. doi: 10.1371/journal.pone.0359272 (PMC13630197; doi:10.1371/journal.pone.0359272)
Supplement: Supplementary file 5 [file pone.0359272.s005.docx]

# **S4 Table. Association between the characteristics of participants who had a COVID-19 infection and diagnostic testing.**

|  | **Diagnosed on PCR / lateral flow test / rapid antigen** | | | | **OR (95%CI)** |
| --- | --- | --- | --- | --- | --- |
|  | **No (n=143)** | | **Yes (n=138)** | |  |
|  | **n** | **(%)** | **n** | **(%)** |  |
| **Age Median [IQR]** | 55 | [52 ;61] | 58 | [53 ;63] | 1.03 (0.99 ; 1.06) |
| **Sex** |  |  |  |  |  |
| Male | 83 | (55.7) | 66 | (44.3) | ref |
| Female | 60 | (45.5) | 72 | (54.5) | 1.51 (0.94 ; 2.42) |
| **Residence** |  |  |  |  |  |
| Inside informal tented settlements | 46 | (54.1) | 39 | (45.9) | ref |
| Outside informal tented settlements | 97 | (49.5) | 99 | (50.5) | 1.20 (0.72 ; 2.00) |
| **Marital status** |  |  |  |  |  |
| Single, divorced or widowed | 38 | (47.5) | 42 | (52.5) | ref |
| Engaged or married | 105 | (52.2) | 96 | (47.8) | 0.83 (0.49 ; 1.39) |
| **Education** |  |  |  |  |  |
| Never attended school | 52 | (47.7) | 57 | (52.3) | ref |
| Elementary | 40 | (48.8) | 42 | (51.2) | 0.96 (0.54 ; 1.70) |
| Preparatory and above | 51 | (56.7) | 39 | (43.3) | 0.70 (0.40 ; 1.22) |
| **Number of chronic conditions** |  |  |  |  |  |
| 0 chronic condition | 33 | (53.2) | 29 | (46.8) | ref |
| 1 chronic condition | 42 | (61.8) | 26 | (38.2) | 0.70 (0.35 ; 1.42) |
| 2 or more | 64 | (43.8) | 82 | (56.2) | 1.46 (0.80 ; 2.65) |
| **Receiving cash assistance** |  |  |  |  |  |
| Yes | 88 | (48.1) | 95 | (51.9) | ref |
| No | 54 | (55.7) | 43 | (44.3) | 0.74 (0.45 ; 1.21) |
| **FIES Food insecurity** |  |  |  |  |  |
| Not food insecure | 7 | (36.8) | 12 | (63.2) | ref |
| Food insecure | 131 | (52.6) | 118 | (47.4) | 0.52 (0.20 ; 1.38) |
| **Household Water Insecurity Experiences Scale** |  |  |  |  |  |
| No household water insecurity | 81 | (47.4) | 90 | (52.6) | ref |
| Household water insecurity | 61 | (56.5) | 47 | (43.5) | 0.69 (0.43 ; 1.12) |
| **Unmet waste need** |  |  |  |  |  |
| No | 113 | (51.1) | 108 | (48.9) | ref |
| Yes | 30 | (50.0) | 30 | (50.0) | 1.05 (0.59 ; 1.85) |
| **Regularization** |  |  |  |  |  |
| No | 107 | (50.0) | 107 | (50.0) | ref |
| Yes | 36 | (53.7) | 31 | (46.3) | 0.86 (0.50 ; 1.49) |
