# Supplementary material for: Predicting COVID-19 infection among older Syrian refugees in Lebanon: A multi-wave survey
Source: PLoS One. 2026 Oct 1;21(10):e0359272. doi: 10.1371/journal.pone.0359272 (PMC13630197; doi:10.1371/journal.pone.0359272)
Supplement: Supplementary file 6 [file pone.0359272.s006.docx]

# **S5 Table: Description of participant behaviors following the COVID-19 infection.**

|  | **n** | **(%)** |
| --- | --- | --- |
| **How many times have you had COVID-19?** |  |  |
| 1 | 262 | (92.6) |
| 2 | 17 | (6.0) |
| 3 | 4 | (1.4) |
| **Diagnosed on PCR or lateral flow test** |  |  |
| No | 143 | (50.9) |
| Yes | 138 | (49.1) |
| **Measures taken the last time you had COVID-19** |  |  |
| Stay at home | 271 | (95.8) |
| Wear a mask | 257 | (90.8) |
| Use traditional remedies (garlic, ginger, anise) | 224 | (79.2) |
| Take medication prescribed by the doctor | 207 | (73.1) |
| Call doctor | 152 | (53.7) |
| Visit healthcare center or doctor | 151 | (53.4) |
| Go to the pharmacist | 124 | (43.8) |
| Take medication on your own without prescription | 61 | (21.6) |
| Call UNHCR | 46 | (16.3) |
| Call MOPH hotline | 16 | (5.7) |
|  |  |  |
| **How long did you stay at home after you found out you had COVID-19? (weeks)** Median [Q1-Q3] | 271 | 3 [2;4] |
