# Supplementary material for: Predicting COVID-19 infection among older Syrian refugees in Lebanon: A multi-wave survey
Source: PLoS One. 2026 Oct 1;21(10):e0359272. doi: 10.1371/journal.pone.0359272 (PMC13630197; doi:10.1371/journal.pone.0359272)
Supplement: Supplementary file 7 [file pone.0359272.s007.docx]

# **S6 Table. Educational level by work for pay during the last 7 days (wave 1).**

| **Education** | **Work status*** | | **P-value**** |
| --- | --- | --- | --- |
|  | **No**  n (%) | **Yes**  n (%) |  |
| Never attended school | 1271 (91.7) | 115 (8.3) | P<0.001 |
| Elementary | 645 (85.9) | 106 (14.1) |  |
| Preparatory and above | 631 (85.2) | 110 (14.8) |  |
| **Total** | **2547 (88.5)** | **331 (11.5)** |  |

*Participants were asked whether they worked for pay during the last 7 days

** There was a statistically significant association between education level and work status (p < 0.001). The proportion of participants who were working increased with educational attainment. Specifically, 8.3% of participants who had never attended school were working, compared with 14.1% of those with elementary education and 14.8% of those with preparatory education or above. An interaction between education level (never attended school vs. elementary education or above) and work status (yes/no) was also tested and found to be statistically significant. Participants with higher educational attainment who were working had higher odds of COVID-19 infection.
