# Supplementary material for: Predicting COVID-19 infection among older Syrian refugees in Lebanon: A multi-wave survey
Source: PLoS One. 2026 Oct 1;21(10):e0359272. doi: 10.1371/journal.pone.0359272 (PMC13630197; doi:10.1371/journal.pone.0359272)
Supplement: Supplementary file 8 [file pone.0359272.s008.docx]

# **S1 Fig. Last occurrence of COVID-19 infection.**
